# High aggressiveness of papillary thyroid cancer: from clinical evidence to regulatory cellular networks

**DOI:** 10.1038/s41420-024-02157-2

**Published:** 2024-08-26

**Authors:** Junsi Zhang, Sunwang Xu

**Affiliations:** 1 Department of Thyroid and Breast Surgery, the First Affiliated Hospital, Fujian Medical University, Fuzhou, China; 2 Department of Thyroid and Breast Surgery, National Regional Medical Center, Binhai Campus of the First Affiliated Hospital, Fujian Medical University, Fuzhou, China; 3 Fujian Provincial Key Laboratory of Precision Medicine for Cancer, Fuzhou, China

**Keywords:** Metastasis, Cancer

## Abstract

The global incidence of thyroid cancer has increased over recent decades. Papillary thyroid cancer (PTC) is the most common type of thyroid cancer and accounts for nearly 90% of all cases. Typically, PTC has a good prognosis. However, some PTC variants exhibit more aggressive behaviour, which significantly increases the risk of postoperative recurrence. Over the past decade, the high metastatic potential of PTC has drawn the attention of many researchers and these studies have provided useful molecular markers for improved diagnosis, risk stratification and clinical approaches. The aim of this review is to discuss the progress in epidemiology, metastatic features, risk factors and molecular mechanisms associated with PTC aggressiveness. We present a detailed picture showing that epithelial-to-mesenchymal transition, cancer metabolic reprogramming, alterations in important signalling pathways, epigenetic aberrations and the tumour microenvironment are crucial drivers of PTC metastasis. Further research is needed to more fully elucidate the pathogenesis and biological behaviour underlying the aggressiveness of PTC.

## Facts

- The incidence of PTC has increased globally in recent years.
- Some PTCs exhibit aggressive behaviours, which significantly increase the risk of postoperative recurrence and metastasis.
- The pathogenesis and regulatory networks involved in the aggressiveness of PTC are complicated and include epithelial-to-mesenchymal transition, tumour cell metabolic reprogramming, signalling pathways and epigenetic modifications.

## Open questions

- What are the characteristics of the high aggressiveness in PTC?
- What are the risk factors increasing the aggressive behaviours of PTC?
- How do these regulatory cellular networks drive the aggressiveness of PTC?

## Introduction

Thyroid cancer is the most common endocrine malignancy, and its incidence has been rapidly increasing globally. In a previous study, the worldwide morbidity, mortality and disability-adjusted life-years of patients with thyroid cancer and the age-standardised incidence rate increased by 60– to 200% from 1990 to 2017 [1]. In China, the age-standardised morbidity of thyroid cancer has tripled in the past decade [2]. Thyroid cancer predominantly affects women; it is the most common malignant tumour in women aged <30 years [3]. In every country, morbidity in women is approximately three times greater than that in men [4]. According to the latest Chinese cancer statistics, thyroid cancer is the fourth most common type of cancer in women in China, with morbidity increasing by 12.4% per year [2, 5].

The majority of thyroid tumours originate from thyroid follicular epithelial cells, whereas 3–5% of thyroid cancers originate from parafollicular cells. Follicular cell-derived cancers can be further subdivided into papillary carcinoma, follicular carcinoma, poorly differentiated carcinoma (insular carcinoma) and anaplastic (undifferentiated) carcinoma [6]. Among them, PTC and follicular thyroid cancer (FTC) are two histological types of differentiated thyroid cancer (DTC) [7]. PTC is the most common type of cancer, accounting for 89.1% of all thyroid cancers and almost all newly diagnosed thyroid cancers [8, 9]. In contrast, medullary thyroid cancer (MTC), anaplastic thyroid cancer (ATC) and poorly differentiated thyroid cancer (PDTC) are rare but highly malignant [10]. According to the cancer statistics of the United States, MTC, which is a rare malignancy, accounts for 1–2% of all thyroid cancers [11]. More than 15–20% of MTC patients develop distant metastases and have a poor prognosis with a 10-year survival of only 10–40% from the time of first metastasis [12, 13]. ATC is another rare subtype of PTC with a morbidity far below six new cases per 100,000 person-years [14]. More than 90% of ATCs exhibit local invasion, 20–50% of ATCs develop distant metastases, the median survival time is less than 5 months, and the one-year survival rate is less than 20% [15]. PDTC has a relatively low incidence rate, accounting for ~2–15% of all thyroid cancers; however, it has a poor prognosis with a five-year disease-specific survival of ~66%. Of PDTC cases, 69% exhibit extrathyroidal extension, and 85% develop distant metastases [15]. These rare subtypes of thyroid cancer exhibit highly malignant phenotypes, but aggressive PTC phenotypes are also commonly observed in the clinic and are poorly understood.

Because the mortality rate for PTC is low and stable, its threat is often overlooked. In fact, PTCs with specific variants or advanced stages exhibit more aggressive characteristics, which may lead to recurrence and metastasis, resulting in an unfavourable prognosis and even death [16]. Notably, some characteristics, such as multifocality and early lymph node metastasis in PTC, are correlated with a recurrence rate as high as 35%, and the 10-year disease-specific survival rate of advanced PTC patients is less than 50% [17, 18]. Because the incidence of PTC accounts for the largest number of thyroid cancers and some variants are aggressive and have a poor prognosis, the aggressiveness of PTC is worth further exploration. However, owing to the lack of a comprehensive understanding of the characteristics, risk factors and molecular mechanisms underlying the metastasis of these aggressive PTCs, the treatment of these patients is inadequate or suboptimal.

Thyroidectomy with radical lymphadenectomy is the major treatment for PTC. However, for late-advanced, metastatic, or recurrent PTC, targeted therapy has become an important adjuvant therapeutic approach. At present, several multikinase inhibitors targeting vascular endothelial growth factor receptor (VEGFR), such as lenvatinib and sorafenib, are licenced by the European Medicines Agency (EMA) and the Food and Drug Administration (FDA) for the clinical treatment of advanced or metastatic PTC [19]. These targeted therapies can cause drug-related adverse events, including hepatic impairment, gastrointestinal symptoms, hypertension, proteinuria, and fatigue, which frequently emerge ~2–3 weeks after the start of medical treatment and result in therapy interruption [10]. Furthermore, there is no general prolongation of overall survival [20–23]. In contrast, surgery for a special subtype of PTC, papillary thyroid microcarcinoma (PTMC), which is characterised by a tumour size of less than 10 mm, is considered overtreatment. In recent years, thyroidectomy has no longer been the first choice for recommended treatment, and active surveillance or delayed surgery have become safe and popular nonsurgical approaches because of the low growth rate of PTMCs [24]. However, some PTMCs still develop lymph node metastasis at the initial diagnostic stages. For PTMCs with aggressive variants or aggressive behaviours, timely surgery should be recommended [25, 26]. Therefore, understanding the aggressive characteristics of PTCs, including late-advanced, metastatic, or recurrent PTCs, which might help us understand more about the oncogenesis of PTC and provide guidance for active surveillance, timely surgery, or even targeted therapy for PTC, is urgently needed.

Here, we describe and analyse PTC metastasis, summarising its aggressive biological behaviour, histopathological features, relative risk factors, and molecular mechanisms of action. This review improves our understanding of the pathogenesis and regulatory networks involved in the aggressiveness of PTC, which will be helpful for the exploration of potential therapeutic strategies, novel biomarkers, and more targeted therapeutic selection.

## Metastatic capacity of PTC

### Characteristics of PTC metastases

**Fig. 1:**
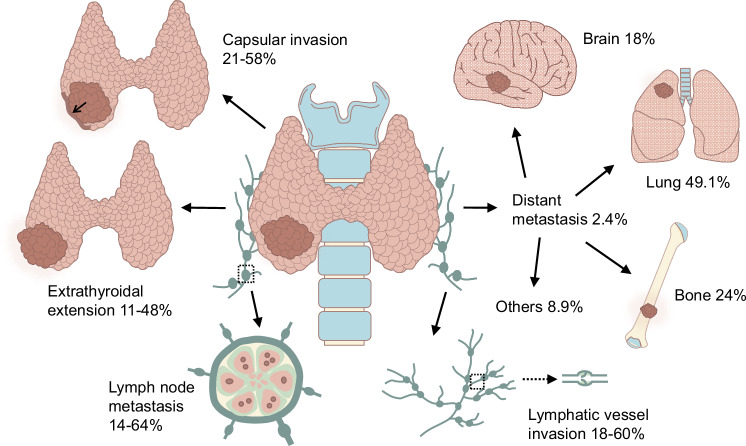
Metastatic capacity of PTC. The common types of metastases in PTC include capsular invasion, which occurs in ~21–58% of cases; extrathyroidal extension (ETE), which occurs in ~11–48% of cases; lymph node metastasis (LNM), which occurs in ~14–64% of cases; and lymphatic vessel invasion (LVI), which occurs in ~18–60% of cases. Distant metastasis (DM) occurs in ~2.4% of all cases, with lung metastasis accounting for 49.1% of DM cases, bone metastasis accounting for 24% of DM cases, and brain metastasis accounting for 18% of DM cases.

Although PTCs grow slowly in situ, capsular invasion, extrathyroidal extension (ETE), and lymph node metastasis (LNM) often occur in practice and are validated by postoperative pathological diagnoses. Distant metastases may be present at the initial diagnosis. The different aggressive behaviours account for the following percentages of cases: capsular invasion, 21–58%; ETE, 11–48%; LNM, 14–64%; and lymphatic vessel invasion (LVI), 18–60% [27–30] (Fig. 1). Patients with capsular invasion are at increased risk of LVI [31]. Mao et al. reported that patients with capsular invasion and ETE had relatively high odds ratios for lymph node metastasis [29]. ETE is also a poor prognostic factor for cancer prognosis and metastasis [32]. Maximal ETE is associated with significantly greater recurrence [33]. In contrast, distant metastases, which account for ~2.4% of cases and include lung, bone, and brain metastases, are associated with a high mortality rate. Lung metastasis is the primary type of distant metastasis [34]. Vuong et al. reported that metastases to the lungs alone were observed in 49.1% of PTCs with distant metastasis [34]. Bone and brain metastases are less common; however, their malignancy should not be underestimated. The incidence of bone metastasis in PTC patients accounts for ~24% of all distant metastases [35]. Furthermore, the 5 and 10-year overall survival (OS) rates after the initial diagnosis of bone metastasis in patients with DTC are reported at 61% and 27%, respectively [36]. Brain metastasis accounts for ~18% of all PTC metastases, with an even worse prognosis and a mean OS ranging from 7.1 to 33 months [37, 38]. Overall, distant metastases are significantly correlated with unfavourable survival outcomes in patients with PTC.

### Lymph node metastasis

LNM is often associated with a high risk of PTC recurrence and is an indicator of poor prognosis. An increase in the N stage has been linked to increased risks of distant metastasis [39]. PTC patients with more than five metastatic lymph nodes have a relatively high recurrence rate [40]. Cervical lymph node metastasis is the most common type of LNM and is considered a vital indicator for deciding the treatment strategy and predicting survival outcomes for patients with PTC. The cervical lymph nodes consist of eight regions, and metastases in the central district are the most prevalent. Delphian lymph node metastasis (DLNM), a subtype of central lymph node metastasis (CLNM), predicts greater aggressiveness and a poor prognosis for PTC [41]. In addition, Yan et al. revealed that DLNM was associated with a higher incidence and greater number of lateral lymph node metastases (LLNMs) as well as an increased likelihood of ETE, lymphovascular invasion and higher rates of central lymph node metastasis (CLNM) [42]. Zhu et al. also demonstrated that DLNM is an indicator of LLNM and distant metastasis [43].

### Pathological features

Based on the pathological findings, PTC can be divided into two subtypes: classical and aggressive. The classical variant has a favourable prognosis, whereas the aggressive variant is associated with poor outcomes. Aggressive variants include diffuse sclerosing variant (DSV), tall-cell variant (TCV), columnar cell variant (CCV), solid variant (SV) and hobnail variant (HV) subtypes [44]. HV is a poorly differentiated cancer cell type, similar to poorly differentiated thyroid cancer (PDTC). The main histological characteristic of DSV is diffuse involvement of one or both thyroid lobes with dense sclerosis, dense lymphocytic infiltrates, abundant psammoma bodies and extensive squamous metaplasia [45]. DSV exhibits aggressive behaviour, such as high rates of vascular invasion, ETE, LNM and distant metastasis [46]. The TCV has a distinctive columnar cell shape, and more than 50% of the cells have a height that is at least three times greater than their width [45]. The WHO defines TCV as a PTC with tall cells comprising more than 30% of the total tumour cells [45]. Furthermore, TCVs frequently exhibit ETE [47]. CCV is histologically defined by the presence of a significant number of columnar cells that display pseudostratified nuclei, the absence of sparse colloids and psammoma bodies [48]. A previous study reported that the CCV is associated with high rates of ETE, LNM and distant metastases [49]. The SV subtypes histologically characterised by the presence of solid, trabecular and insular nests. SVs are also associated with a large tumour size and high rates of LVI, LNM and extracapsular infiltration [47]. In contrast, HV is characterised by 'hobnail' cells (i.e. expanded tumour nuclei, bulging from the surface of the epithelium), a micropapillary structure, a high nuclear/cytoplasmic ratio, loss of cellular polarity and intranuclear inclusions and nuclear grooves [44, 50]. ETE (58.3%), LVI (41.7%) and LNM (75%) [51] are frequently found in HVs. Overall, patients with aggressive PTC variants exhibit poorer disease-free survival (DFS), indicating that aggressive variants are associated with higher rates of recurrence and metastasis [44, 52].

### Summary of high aggressiveness of PTC

In conclusion, the pathological characteristics, metastatic ability and pathological characteristics of PTC variants determine their malignant behaviour and prognosis. Therefore, we define the high aggressiveness of PTC as follows: 1) PTC with capsular infiltration or ETE, 2) CLNM or distant metastasis and 3) aggressive variants in pathological subtypes.

## Risk factors for PTC aggressiveness

### Age

**Fig. 2:**
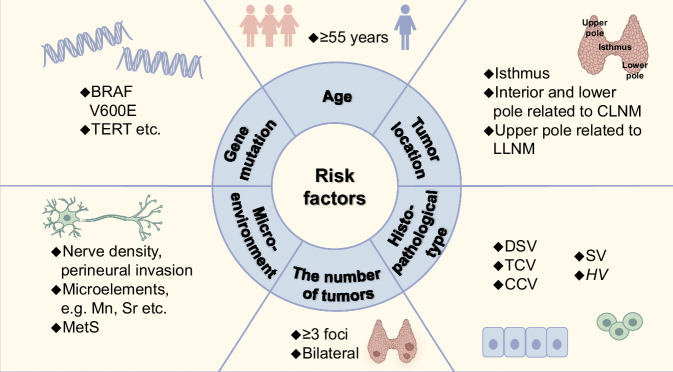
Risk factors for high aggressiveness of PTC. Approximately six risk factors are associated with high PTC aggressiveness. (1) Age ≥55 years old. (2) The tumours are located in the isthmus and different locations are associated with different types of LNM. (3) The patients with ≥3 foci or bilateral. (4) Microenvironment, which includes high nerve density, perineural invasion, microelements, and MetS. (5) Uncommon histopathological types, such as DSV, TCV, CCV, SV, and HV. (6) Gene mutation, such as BRAF V600E and TERT mutation.

Age ≥ 45 years was used as a prognostic marker of PTC according to the 7th Edition of the AJCC cancer staging manual. However, in the 8th Edition, the age cut-off was revised to ≥55 years. Kaliszewski et al. reported that patients ≥55 years of age present a greater number of aggressive features, including capsular invasion, ETE and LNM, as well as a higher risk of locally advanced stages compared with patients <55 years of age [53]. Notably, a recent study indicated that paediatric and young adult patients with PTC, particularly those under 30 years of age, have a greater risk of LNM, nodal disease and lateral neck metastases [54]. However, the underlying cause of the increased risk of LNM and lateral neck metastases in younger patients remains unclear. Menno et al. reported that the difference in LNM incidence between younger and older patients with PTC could be attributed to the activity of six candidate genes (ECM1, ERBB2, UPA, PFKFB2, MEIS2 and CA2) [55]. Additionally, a multicentre retrospective study revealed that the levels of protective immune cells, such as plasma cells, resting mast cells and resting natural killer cells, were significantly decreased in children and young adults, thereby contributing to the more aggressive nature of PTC in this age group [56]. Further studies are needed to understand the detailed mechanisms underlying these phenomena (Fig. 2).

### Tumour location

Tumour location is associated with aggressiveness. Tumours located in the isthmus may have a greater risk of aggressive behaviours such as ETE and CLNM. Tumours located in the interior and lower poles of the thyroid are more likely to metastasise to the central lymph nodes, whereas those in the upper pole have a greater risk of LLNM, often skipping lateral cervical metastasis [57, 58]. The middle posterior lateral, inferior anterior central and inferior posterior lateral nodules are associated with CLNM [58] (Fig. 2).

### Multifocality

A previous study revealed that the risk of aggressive clinicopathological features in PTC patients increased positively with the number of tumours [59]. The presence of multiple foci was found to be associated with a high frequency of LNM, ETE and vascular invasion, particularly in patients with three or more tumour foci. Multifocality has been identified as an independent risk factor for the recurrence of various variants of PTC, including classic PTC, follicular variant PTC, TCV and PTMC. In addition, bilateral PTC is associated with higher rates of ETE and CLNM [60] (Fig. 2).

### Histopathologic features

Compared with the classic subtypes, the uncommon subtypes DSV, TCV, CCV, SV and HV are more aggressive. These rare subtypes have a greater risk of poor prognosis and account for 0.7–6.6%, 1–19%, 0.15–0.2%, 1–3%, and 1.08% of PTCs, respectively [50, 61–64]. In a study by Limberg et al. in patients with invasive features, TCV, CCV and DSV were independently associated with worse overall survival compared with classic PTC [65]. Additionally, Allen et al. reported that patients with SV and HV had approximately double the risk of death compared with those with DSV or TCV. Remarkably, a high distant metastasis rate was observed at initial presentation in the HV and SV subtypes (16.1–24.3%), which was higher than that in the DSV (2.3%) and TCV subtypes (4.0%). In other words, patients with HV or SV may have a worse prognosis [9] (Fig. 2).

### Tumour microenvironment

The tumour microenvironment (TME) is an important driving factor in tumour progression. Among TME components, nerves play a role as regulatory factors. Rowe et al. demonstrated that nerve density was significantly greater in PTC than in benign thyroid tissues. Nerve density and perineural invasion were positively associated with extrathyroidal invasion [66].

The tumour interstitial fluid is another component of the TME. Several studies have confirmed that microelements in the TME are strongly correlated with risk factors for highly aggressive PTC. Patients with PTC with higher urinary levels of Mn and Sr and lower urinary levels of Fe, Co, and Mo exhibited significantly aggressive characteristics. Patients with PTC and high urinary levels of Mn had a greater risk of capsular invasion and advanced T stage. Similarly, high urinary Sr levels increase the risk of multifocality and advanced T stages (T3/4a/4b). Conversely, low urinary Fe levels increased the risk of large tumour size (1 cm), capsular invasion and advanced T stage (T3/4a/4b). Similarly, low urinary levels of Co and Mo increase the risk of capsular invasion and lymph node metastasis, respectively [67]. Metabolic syndrome (MetS) is associated with PTC aggressiveness. MetS represents a cluster of metabolic abnormalities, including central obesity (waist circumference ≥90 cm in Chinese men and waist circumference ≥85 cm in Chinese women), hyperglycaemia (fasting glycaemia >6.1 mmol/L or previous diagnosis of type 2 diabetes), high blood pressure (≥130/85 mmHg or previous diagnosis of hypertension) and dyslipidaemia (TG concentration ≥1.70 mmol/L, HDL concentrations <1.04 mmol/L) [68, 69] (Fig. 2).

### Gene mutations

Common PTC mutations include B-Raf proto-oncogene serine/threonine kinase (BRAF) and telomerase reverse transcriptase (TERT) mutations [6]. Among these, BRAF V600E is the most prevalent mutation, ranging from 25% to 82% [70]. Silver et al. demonstrated that the BRAF V600E mutation is associated with aggressive behaviour in PTC, including ETE, LNM, advanced stage and recurrence [71]. The underlying molecular mechanism is that tumours driven by BRAF V600E exhibit high extracellular signal-regulated kinase phosphorylation, which activates the mitogen-activated protein kinase (MAPK) signalling pathway, leading to unregulated cell proliferation and a poor prognosis [72].

Additionally, ~7.5% of PTCs harbour TERT mutations [73]. TERT is a catalytic subunit of telomerase that cooperates with integral RNA subunits and several species-specific assessor proteins to add telomeres [74]. TERT maintains the length of telomeres at the ends of chromosomes to maintain cellular functions, such as proliferation and cell cycle progression [75]. Mutations in the TERT promoter activate TERT transcription, which is responsible for the origination of PTCs arising from differentiated cell types. PTCs with TERT promoter mutations have higher rates of extrathyroidal and vascular invasion, LNM, distant metastasis and recurrence (Fig. 2).

### Protective factor: Hashimoto’s thyroiditis

In contrast, Hashimoto’s thyroiditis (HT), the most common autoimmune disease in humans, has been suggested to be a protective factor against the aggressiveness of PTC, and HT is associated with a lower incidence of aggressive characteristics and a better prognosis in PTC patients [76]. Although patients with HT had a greater average number of lymph nodes resected from the central neck than those with PTC alone did, they were less likely to have central and lateral lymph node metastases. Moreover, these patients had a lower risk of primary tumours ≥4 cm, ETE, vascular invasion, recurrence, and distant metastasis. Therefore, patients with PTC and HT have a better prognosis [77, 78].

## Potential mechanisms

### Epithelial-to-mesenchymal transition

**Fig. 3:**
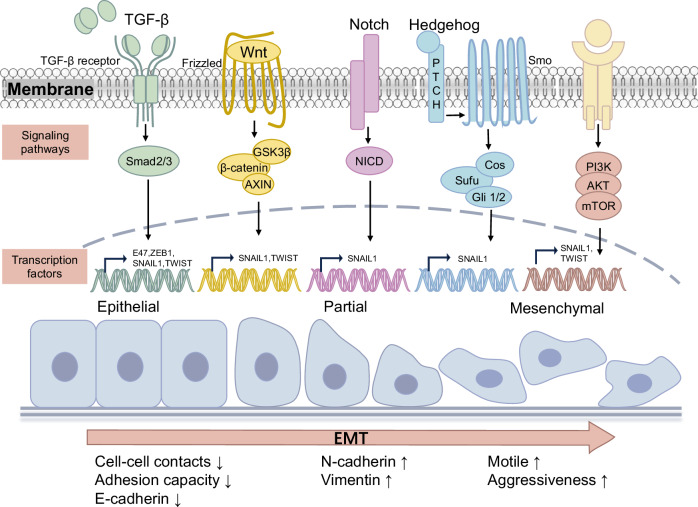
Signalling pathways and transcription factors in activating EMT of PTC. EMT is a cellular programme that transforms epithelial cells into mesenchymal cell states. During PTC metastasis, multiple EMT-inducing transcription factors (EMT-TFs) trigger the signalling pathways to modulate EMT. In this process, epithelial cells gradually lose their cobblestone epithelial appearance and convert into a spindle-shaped, mesenchymal morphology, along with losing cell-cell contacts and adhesion capacity. The E-cadherin levels decrease, while N-cadherin and Vimentin levels increase. As a result, tumour cells become more mobile and aggressive.

The epithelial-to-mesenchymal transition (EMT) is a cellular process that occurs naturally during development [79]. This reversible cellular programme converts epithelial cells into mesenchymal cells. During this process, epithelial cells gradually lose their cobblestone epithelial appearance in monolayer cultures, along with cell-cell contact and adhesion capacity, and convert into a spindle-shaped mesenchymal phenotype that is more motile [80, 81]. E-cadherin levels decrease, whereas N-cadherin and vimentin levels increase, enhancing the motility of tumour cells. EMT can facilitate the aggressiveness of tumour cells [82] (Fig. 3).

#### EMT-related signalling pathways

In recent years, EMT has been considered a potential mechanism contributing to the increased metastasis of PTC cells, and it can be induced by a variety of intracellular signalling pathways. When epithelial cells receive specific signals released by the cells that constitute their stromal microenvironment, these signalling pathways can be activated [83]. The interplay and reciprocal effects between different signalling pathways also influence the initiation and progression of EMT (Fig. 3).

##### TGF-β

The transforming growth factor-β (TGF-β) signalling pathway plays a critical role in initiating EMT. In PTC, sineoculis homeobox homologue 1 (SIX1) promotes this pathway, facilitating the acquisition of motility and migratory abilities by PTC cells to penetrate the basement membrane and invade adjacent tissues, eventually resulting in distant metastases [84].

##### Wnt

The Wnt signalling pathway is also involved in PTC progression. Tripartite motif 44 (TRIM44), a member of the TRIM family, is highly expressed in PTC. TRIM44 promotes the activation of the Wnt/β-catenin signalling pathway, inducing EMT and facilitating the proliferation, migration and aggressiveness of PTC cells [85]. Additionally, DOCK9 antisense RNA2 (DOCK9-AS2), an exosomal lncRNA derived from PTC cancer stem-like cells (PTC-CSCs), is highly expressed in PTC [86]. Mechanistically, DOCK9-AS2 enhances β-catenin expression via SP1 and miR-1972 to activate the Wnt/β-catenin pathway, inducing EMT and enhancing the progression of PTC [86].

##### Notch

In many studies, the Notch pathway has been implicated as a driver of EMT. Downregulation of miR‐599 may induce EMT by activating the Notch signalling pathway and upregulating HEY2, thereby promoting the proliferation, migration and aggressiveness of PTC [87].

##### Hh

The hedgehog (Hh) signalling pathway is a vital mediator of PTC cell aggressiveness and metastasis. Low miR-431 expression activates the Hh signalling pathway by upregulating Gli1 expression, thereby facilitating EMT. During this process, the epithelial marker E-cadherin decreases, whereas the mesenchymal marker vimentin increases, thereby promoting PTC metastasis [88].

##### PI3K/AKT

The phosphoinositide 3-kinase (PI3K)/AKT signalling pathway can also induce EMT. Nectin cell adhesion molecule 4 (NECTIN4), a member of the NECTIN family, a group of Ca^2+^-independent immunoglobulin-like molecules, is significantly overexpressed in PTC and modulates EMT by activating the PI3K/AKT signalling pathway to drive PTC metastasis [89, 90].

#### EMT-inducing transcription factors (EMT-TFs)

In this section, we discuss various EMT-TFs that activate signalling pathways to modulate EMT during PTC progression, including SNAIL1, the basic helix–loop–helix factor TWIST and the zinc finger E-box-binding homeobox factors ZEB1 and E47.

The regulation of SNAIL1 and TWIST in PTC has been well documented. DDR2 overexpression induces EMT and promotes the metastasis and aggressiveness of PTC cells. This process is dependent on the upregulation of the SNAIL1 protein and the activation of extracellular signal-regulated kinase (ERK) 2 [91], whereas high expression of Sirtuin 6 (SIRT6) increases hypoxia-inducible factor-1α (HIF-1α) to regulate the expression of both SNAIL and TWIST. HIF-1α can directly bind to the hypoxia-response element (HRE) in its promoter to upregulate SNAIL and TWIST. The overexpression of SNAIL and TWIST downregulates E-cadherin and upregulates vimentin to promote EMT and PTC progression [92, 93].

ZEB1, another EMT-related TF, can inhibit cadherin 1 (CDH1) transcription and recruit other chromatin-modifying factors to their promoters, inducing the expression of genes encoding N-cadherin and vimentin and promoting EMT [83]. ABC transporter A1 (ABCA1), a member of the ABC subfamily A, is involved in the reverse cholesterol transport pathway (RCT), which is crucial for regulating cellular cholesterol, phospholipid efflux and lipid homoeostasis [94]. The overexpression of ABCA1 promotes ZEB1 transcription by activating the ERK/Fra-1 pathway. This process is thought to drive PTC lung metastases [95]. Furthermore, taurine-upregulated gene 1 (TUG1), a lncRNA located on chromosome 22q12, is upregulated in PTC [96, 97]. Elevated expression of TUG1 can promote the EMT, proliferation, metastasis and aggressiveness of PTC by competitively sponging miR-145 to upregulate ZEB1 [97].

The upregulation of E47, an alternative splice variant of the E2A gene, also enhances the metastasis of PTC through the induction of EMT by binding to the E-box elements on the promoter region of CDH1 and inhibiting its transcription [98, 99]. Inhibitor of DNA-binding 3 (ID3) exists within a subset of helix–loop–helix transcription factors that have lost their DNA-binding region. Upregulated ID3 interacts with E47 to decrease its interaction with the promoter region of CDH1 and prevent the repressive effects of E47 on CDH1 transcription, maintaining E-cadherin transcription and the epithelial phenotype of PTC [99].

### Tumour cell metabolic reprogramming

Compared with normal cells, cancer cells are surrounded by completely different microenvironments that contribute to cancer progression and invasion. In response to hypoxia and a low-nutrient microenvironment, tumour cells must adapt [100]. The metabolic processes of cancer cells have been modified to enable uncontrolled proliferation [101]. This phenomenon is known as “metabolic reprogramming”, which is necessary for malignant tumour progression, including aggressiveness and metastasis. Metabolic reprogramming is an emerging concept providing associated therapeutic strategies, and it is considered an important hallmark of PTC [100].

#### Glycolysis

Glycolysis is the most well-known metabolic process exploited in tumour cells through reprogramming, and hypoxia is a crucial microenvironmental condition that activates this process [102]. To adapt to hypoxic conditions, tumour cells switch their glucose metabolism from oxidation to anaerobic glycolysis, resulting in increased glucose uptake, lactate production, and ATP generation. This phenomenon is known as the “Warburg effect” [103–105].

Lactate dehydrogenase A (LDHA) is a critical enzyme that catalyses the final step of glycolysis [106, 107]. Two novel upstream regulators of LDHA, STAT and LINC00671, have been identified to have close association with glycolysis in PTC. Hypoxia primarily stimulates the transcription factor STAT3 to suppress LINC00671 expression, activate LDHA expression, facilitate the Warburg effect, and promote PTC invasion and metastasis [108].

FAM111B, located on human chromosome 11q12.1, encodes a protein characterised by a trypsin-like serine/cysteine peptidase domain at the C-terminus [109]. FAM111B negatively regulates PTC cell glycolysis and progression. However, oestrogen increases the recruitment of DNMT3B to the promoter region of FAM111B and facilitates DNMT3B-mediated CpG methylation of the FAM111B promoter to suppress the expression of FAM111B, thus promoting glycolysis and the progression of PTC. Consequently, the downregulation of FAM111B predicts a worse prognosis in patients with PTC [110].

Hypoxia-inducible factor 1α (HIF-1α) activates various genes involved in glycolysis to positively regulate the Warburg effect. These genes include glucose transporter 1 (GLUT1), which has hypoxia-response elements (HREs) in its promoter [111].

Yes-associated protein (YAP) is usually overexpressed in PTC and is associated with ETE and distant metastasis [112–114]. YAP has also been demonstrated to upregulate the expression of genes related to glycolysis and glucose transporters, such as GLUT1, which is also regulated by HIF-1α, thus impacting glucose metabolism [115, 116]. Song et al. demonstrated that hypoxia induces YAP activation, leading to interaction with HIF-1α to form a YAP/HIF-1α complex that maintains the protein stability of HIF-1α and activates GLUT1 transcription, thereby directly accelerating glycolysis and the progression of PTC [102].

#### Acetyl-CoA

Acetyl-coenzyme A (acetyl-CoA) is an essential metabolite in glucose metabolism. Oxidative phosphorylation of acetyl-CoA fuels the mitochondrial TCA cycle to produce ATP. Acetyl-CoA participates in histone 3 lysine 27 acetylation (H3K27ac), a widely known epigenetic mechanism. LDHA also participates in glucose metabolism. It converts pyruvate and NADH to lactate, catalysing the final step of glycolysis and contributing to the Warburg effect. This creates an acidic tumour microenvironment that is beneficial for EMT and metastasis [117–119]. In PTC, the upregulation of LDHA promotes acetyl-CoA production. High expression of acetyl-CoA increases histone H3K27ac, accelerating EMT and leading to the progression and invasion of PTC [119]. LDHA links glucose metabolism to chromatin remodelling via epigenetics.

#### O-GlcNAcylation

O-GlcNAcylation is a posttranslational modification (PTM) that can be activated by β-N-acetylglucosaminyl transferase (O-GlcNAc transferase, OGT) [120]. In PTC cells, high OGT expression induces increased O-GlcNAcylation of YAP [112]. YAP is widely considered a key element of tumour suppressor pathways such as the Hippo pathway [121, 122]. Downstream of the Hippo pathway, the transcriptionally enhanced associated domain (TEAD) serves as the final nuclear effector, and its target genes regulate tumour cell progression [123]. When the Hippo pathway is inactivated, activated YAP enters the nucleus, where it functions as a transcriptional coactivator of TEAD and enhances gene expression [121–125]. OGlcNAcylated YAP inhibits YAP Ser127 phosphorylation to prevent its cytoplasmic localisation and protein degradation, which enhances the stability of YAP. Consequently, YAP translocates from the cytoplasm to the nucleus, where it binds to TEAD and stimulates downstream gene transcriptional activation, eventually increasing tumour aggressiveness [112].

### Altered key signalling pathways

#### PI3K/AKT

Dysregulation of the PI3K/AKT axis has been well documented in PTC [126]. This pathway is responsible for transmitting signals from various cell membrane receptor tyrosine kinases to the nucleus, where they regulate multiple cellular processes, including proliferation, differentiation and survival [6]. PI3K/AKT signalling is associated with cell proliferation, apoptosis, and autophagy. Circ_PSD3 acts as a molecular sponge for miR-637, subsequently upregulating HEMGN and increasing PI3K/AKT pathway activity. This process plays a vital role in promoting PTC development, cell cycle progression, proliferation and motility while inhibiting apoptosis [126].

The tektin4 gene (TEKT4), an oncogene belonging to the TEKT family localised on chromosome 2, encodes a constitutive protein of microtubules in cilia, flagella, basal bodies and centrioles. These compounds can promote cell proliferation, colony formation, migration and invasion. High TEKT4 expression facilitates PTC tumorigenesis by enhancing the activity of the PI3K/Akt signalling pathway [127].

**Fig. 4:**
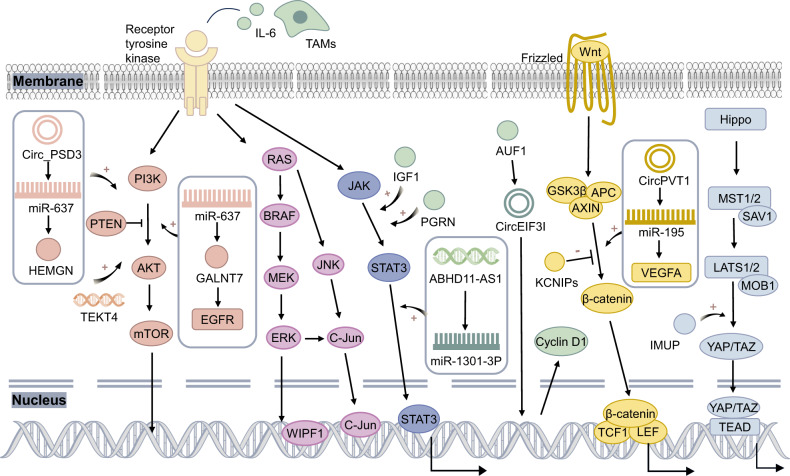
Multiple signalling pathways drive the aggressiveness of PTC. The molecular mechanism of pathogenesis in PTC involves multiple signalling pathways that form a network. It includes the dysregulation of the phosphatidylinositol-3 kinase (PI3K)/AKT, mitogen-activated protein kinase (MAPK), JAK/STAT3, Wnt/β-catenin and Hippo pathways. Various nucleic acids and protein molecules activate these signalling pathways, which in turn contribute to the aggressiveness of PTC.

The overexpression of miR-30b-5p significantly suppresses PTC cell proliferation, migration and invasion while promoting apoptosis. However, the upregulation of GALNT7 has been shown to promote the proliferation and metastasis of PTC. GALNT7 neutralises the effect of miR-30b-5p on PTC metastasis. Mechanistically, miR-30b-5p suppresses the progression and metastasis of PTC by targeting GALNT7 and subsequently inhibiting the EGFR/PI3K/AKT pathway [128] (Fig. 4). PI3K/AKT is also an important metastasis-related signalling pathway in ATC and PDTC, and PIK3CA mutation contributes to the recurrence and metastasis of ATC and PDTC [129]; however, whether PI3K/AKT is a common aggressive driver of metastasis in all subtypes of thyroid cancer needs to be further investigated.

#### MAPK

Another molecular signalling pathway that participates in the regulation of PTC development is the MAPK axis. The MAPK pathway plays a crucial role in regulating various cellular functions, such as growth, proliferation, apoptosis and metabolism, by modulating the expression of multiple genes [130]. BRAF, a cytoplasmic serine–threonine protein kinase, plays a pivotal role in cell signalling by activating the MAPK pathway. Notably, the V600E transversion is the most common mutation in BRAF [131], which contributes to the oncogenesis and aggressiveness of PTC by excessively activating the MAPK pathway [130].

WASP-interacting protein family member 1 (WIPF1), a widely expressed proline-rich multidomain protein, is an oncogene that drives the aggressiveness of cancer [132, 133]. BRAF V600E can activate the MAPK pathway and trigger hypomethylation of the WIPF1 promoter, leading to the overexpression of WIPF1, thereby promoting the invasion of PTCs, such as LNM and ETE [134]. Additionally, the BRAF V600E mutation can upregulate BRAF-activated nonprotein-coding RNA (BANCR) and activate the MAPK pathway [135]. In addition, IL-6 produced by M2 macrophages can activate the MAPK and JAK-STAT3 signalling pathways, resulting in increased expression of the transcription factors p-c-Jun and p-STAT3, which can bind to the PD-L1 promoter to activate PD-L1 transcription and contribute to the aggressiveness of PTC [136] (Fig. 4). In addition, the activated MAPK signalling pathway is associated with the aggressive phenotypes of FTC, PDTC and ATC [137], but the underlying mechanisms are unclear.

#### STAT3

STAT3 signalling is important for driving tumour growth, migration, angiogenesis and inflammatory crosstalk with immune cells during carcinogenesis [138, 139]. As mentioned above, in tumour cell metabolic reprogramming, the STAT3/LINC00671/LDHA axis regulates glycolysis, growth and metastasis in PTC [108]. In addition, insulin-like growth factor-1 (IGF1) is overexpressed in PTC and promotes PTC progression and invasion via the STAT3 signalling pathway [140]. Progranulin (PGRN), a glycoprotein secreted by various epithelial cells, has significant implications for inflammatory mechanisms and tumour progression. In PTC, high PGRN expression facilitates cell proliferation, accelerates cell cycle transition, inhibits apoptosis and drives metastasis by activating the JAK2-STAT3/4 signalling pathway [141]. High expression of ABHD11-AS1 positively regulates the PI3K/AKT signalling pathway and sponges miR-1301-3p to upregulate the STAT3 pathway, thereby promoting the development of PTC [142] (Fig. 4).

#### Other pathways

Other molecular signalling pathways are also involved in the regulation of PTC aggressiveness. For example, low expression of Kv channel interacting protein 3 (KCNIP3) can activate the Wnt/β-catenin signalling pathway and regulate EMT, subsequently promoting the aggressiveness of PTC [143]. CircPVT1 can promote the expression of VEGFA by sponging miR-195 and activating the Wnt/β-catenin signalling pathway, thus contributing to the malignant progression and invasion of PTC [144]. Immortalisation upregulated protein (IMUP) is highly expressed in PTC. IMUP promotes cell proliferation, progression, and metastasis through the Hippo–YAP1 pathway [145]. Further studies are needed to elucidate the mechanisms of other molecular pathways involved in PTC carcinogenesis (Fig. 4).

### Epigenetic alterations

Epigenetics are heritable modifications of cellular phenotypes that occur independently of changes in DNA sequences. Epigenetic alterations include DNA methylation, histone acylation, chromatin accessibility and noncoding RNA regulation, and these alterations regulate numerous biological processes that play crucial roles in the development of cancer [146].

#### DNA methylation

DNA methylation regulates gene expression by attaching a methyl group to the fifth carbon of the cytosine residues in CpG dinucleotides. Clusters of CpG dinucleotides, called CpG islands, are generally located in promoter regions [147]. Differential methylation has been observed in the genes of several oncogenic pathways in PTC [148, 149]. The PTC genome is globally hypomethylated, and most oncogenic signalling pathways are hypomethylated [148]. Hypermethylation can induce the downregulation of tumour suppressor genes, such as NIS, RAR2 and TIMP3, to affect tumour dedifferentiation and metastasis [148]. Conversely, carcinogenic genes such as proto-oncogenes are upregulated by hypomethylation [147]. Overexpression of solute carrier family 34 member A2 (SLC34A2), which is mediated by hypomethylation, promotes PTC cell progression, migration and aggressiveness, has been verified to be associated with the regulation of the PTEN/AKT/FOXO3a pathway [150]. Moreover, proto-oncogene transmembrane 4 superfamily 1 (TM4SF1) is highly expressed in PTC and is associated with DNA promoter hypomethylation [151]. Zhao et al. demonstrated that the tumour suppressor gene NDRG4 is silenced in PTC because of its DNA promoter hypermethylation. This epigenetic mechanism of DNA methylation illustrates the activation of oncogenes in tumours. For example, DNA promoter hypomethylation-mediated transcriptional activation of the proto-oncogenes FOXO3, ZEB2 and CDK6 can drive lymph node metastasis and invasion in PTC [152].

#### Histone acetylation

Histone acetylation plays a crucial role in epigenetics, affecting the proliferation and development of PTC cells and influencing the prognosis of patients with PTC through the modulation of histone acetylation via histone acetylases and histone deacetylases [153]. In epigenetic transcriptional regulation, histone acetylation alters the chromatin structure, thereby influencing gene transcription. Conversely, histone deacetylation silences genes by obstructing the accessibility of transcription factors to the binding sites on gene promoters [154].

H3K27ac is a well-known indicator of transcriptional activation. Zhang et al. reported that H3K27ac is widely distributed in intronic and intergenic regions throughout the PTC genome, where it regulates genes involved in cell activation, cell-cell adhesion, and the secretion of chemical substances [155]. Superenhancers (SEs) play important roles as genetic transcriptional regulators in PTC. SEs are defined as clusters of enhancers densely occupied by mediators and chromatin regulators and are characterised by high levels of H3K27ac. Transcription factors bind to SEs and activate transcription [156, 157]. In PTC, activated SEs modulate the high expression of proto-oncogenes, such as ALOX5, ELF3 and PLXNC1, which are related to tumour invasion. Conversely, inactivated SEs modulate the low expression of suppressor genes, such as SLCO2A1 and ARHGAP24 [155].

In addition, crosstalk between DNA demethylation and histone acetylation in PTC facilitates gene expression, resulting in tumour development. In our previous study, we reported that NR4A1 directly binds to the promoter region of LEF1, resulting in crosstalk between histone acetylation and DNA demethylation. This interaction leads to the transcriptional upregulation of LEF1 expression, subsequently facilitating the expression of downstream growth-related genes in PTC [158].

#### Chromatin accessibility

Chromatin accessibility refers to the degree to which nuclear macromolecules come in physical contact with chromatinized DNA. Accessibility is determined by the occupancy and topological organisation of nucleosomes as well as other chromatin-binding factors that impede access to DNA. Chromatin accessibility is a dynamic mechanism in the epigenome, establishing a collection of potential regulatory sites throughout the genome. The arrangement of open chromatin across enhancers, promoters, and gene bodies provides a flexible template through which components of the chromatin epigenome interact [159].

The SWI/SNF complexes comprise 12–15 subunits that hydrolyse ATP to mobilise nucleosomes and remodel chromatin during assembly. They primarily act on enhancers located distal to the transcription start sites of genes and are linked to developmental processes [160]. The subunits of the SWI/SNF chromatin-remodelling complexes frequently undergo mutations in advanced PTC [161]. The absence of these subunits, including Arid1a, Arid2 and Smarcb1, results in a general reduction in chromatin accessibility, followed by the differentiation of PTCs [161]. In addition, the absence of Arid1a, Arid2, or Smarcb1 downregulates the expression of transcription factors (TFs), such as PAX8, NKX2-1 and FOXE1 [161, 162]. These TFs are necessary for PTC development [163]. In general, the loss of subunits from SWI/SNF chromatin-remodelling complexes decreases chromatin accessibility, which suppresses transcription. This process is associated with the development and invasion of PTC [161].

Intriguingly, there are some differences in chromosome number variation among PTCs and other types of thyroid cancer. The genome of PTC predominantly exhibits a diploid nature, whereas in PDTC and ATC, alterations in chromosome copy number are prevalent and tend to occur more frequently in tumours devoid of driver gene mutations [164]. The presence of gene rearrangements that are commonly observed in PTC, such as RET/PTC and PAX8-PPARγ, are only seen in ~14% of PDTC cases, and they are absent from ATC [164]. Therefore, these differences in chromosomes might also explain the different levels of aggressiveness and prognoses between PTC and other rare types of thyroid cancer.

#### Noncoding RNAs

Noncoding RNAs are mostly clusters of RNAs without obvious protein-encoding functions. However, recent studies have shown that some variants of noncoding RNAs, such as long noncoding RNAs (lncRNAs) and circular RNAs (circRNAs), can encode micropeptides in mammalian cells [165]. According to size and shape, noncoding RNAs can be divided into small RNAs, such as microRNAs (miRNAs); long noncoding RNAs (lncRNAs); and circular RNAs (circRNAs).

##### miRNAs

miRNAs are a class of small noncoding regulatory RNAs 17–25 nucleotides in length. miRNAs are generated by Dicer, an RNase that processes hairpin-structured precursors (pre-miRNAs) into mature miRNAs [166]. In general, miRNAs recognise and bind to the 3’-untranslated region (3’-UTR) of target mRNAs to repress the expression of the target gene at the posttranscriptional level [167]. miRNAs play critical roles in the regulation of cancer biology, including the cell cycle, programmed cell death, cell invasion and metastasis, and angiogenesis, to drive tumour initiation and development [167].

In PTC cells, miR-335-5p expression is inhibited. miR-335-5p targets the 3’-UTR and decreases expression of intercellular adhesion molecule 1 (ICAM‑1) mRNA, an oncogene protein that drives the metastasis of tumour cells by recruiting inflammatory cells and promoting the proliferation, angiogenesis and invasion of cancer cells [168]. Pellino-1 (PELI1), a novel cancer-related E3 ubiquitin ligase, is expressed in various cancers [169]. Loss of miR-30c-5p induces an increase in PELI1 and then activates the PI3K/AKT signalling pathway, leading to PTC cell proliferation and migration [169].

##### lncRNAs

lncRNAs are a class of transcripts that are more than 2000 nucleotides in length that cannot be translated into proteins [170]. lncRNAs can regulate chromatin dynamics, gene expression or protein stability to modulate tumour cell growth, differentiation and development [171].

lncRNAs also participate in the generation of aggressive phenotypes of PTC. For example, the expression of the lncRNA SLC26A4-AS1 is significantly decreased in PTC, and this is associated with the increased expression of multiple DNA double-strand break (DSB) repair genes, especially genes encoding proteins in the MRE11/RAS50/NBS1 (MRN) complex [170]. In addition, SLC26A4-AS1 can simultaneously interact with DDX5 and the E3 ligase TRIM25, thereby enhancing the degradation of DDX5 via the ubiquitin-proteasome pathway. This process enhances the ability of PTC cells to invade and metastasise [170]. HOTAIR (HOX transcript antisense intergenic RNA), a well-known lncRNA, sponges miR-1 and induces CCND2 expression or interacts with polycomb repressive complex 2 (PRC2) or lysine-specific histone demethylase 1 (LSD1) to regulate H3K27 trimethylation and H3K4 demethylation, respectively [172, 173]. Moreover, HOTAIR can inhibit DLX1 expression via the modulation of histone modifications [174]. As a consequence, the lncRNA HOTAIR promotes the proliferation, migration and invasion of PTC.

##### circRNAs

circRNAs possess a closed continuous loop structure, and they are generated primarily from the back-splicing of precursor mRNAs (pre-mRNAs) and rarely from the self-splicing introns of ribosomal RNAs, mitochondrial RNAs and tRNAs; they may or may not have protein coding ability [175–177]. According to their origins, circRNAs can be divided into circular intronic RNAs (ciRNAs), exotic circRNAs (EciRNAs) and exon–intron circRNAs (EIciRNAs) [178]. Recent research indicates that circRNAs play essential roles in the regulation of tumour cells by functioning as activity regulators. They act as miRNA sponges, agents that interact with proteins, or translation templates to activate or inhibit the progression of tumour cells [175, 179].

In PTC, circEIF3I interacts with AU-rich element (ARE) RNA-binding factor 1 (AUF1) to increase Cyclin D1 mRNA stability and increase its translation, resulting in the promotion of PTC progression [180]. In addition, circPRKCI functions as a sponge for miR-1301-3p to modulate the expression of HMGB1, which regulates transcription and DNA organisation in the nucleus [181]. Circ_0000644 sponges miR-671-5p, increasing annexin A2 (ANXA2) expression, resulting in the promotion of PTC malignancy [182]. Together, these findings indicate that noncoding RNAs are also key drivers that promote the development of aggressive and malignant behaviours in PTC.

### Tumour microenvironment

#### Innervation

The TME, which consists of a variety of cellular and noncellular components, is essential for the invasion and progression of PTC [175]. Both nerve density and perineural invasion are associated with the aggressiveness of PTC. The mechanism may involve proNGF/NGF, the ligand of the tyrosine kinase receptor TrkA, which stimulates nerve terminal outgrowth via the TrkA signalling pathway in the PTC microenvironment [66].

**Fig. 5:**
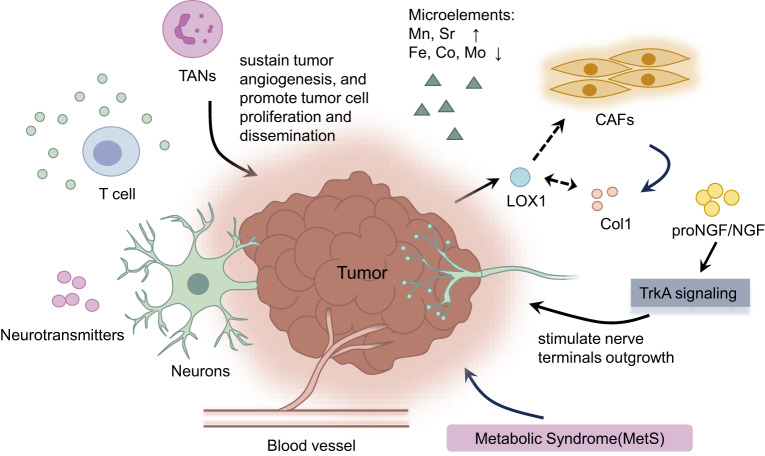
Influence of the tumour microenvironment on the aggressiveness of PTC. The tumour microenvironment (TME), which consists of a variety of cellular and non-cellular components, is an essential factor in the aggressiveness of PTC. Nerve density and perineural invasion modulate the TME. Cancer-associated fibroblasts (CAFs) and tumour-associated neutrophils (TANs) infiltrate in TME, which are associated with tumour cell motility, progression, proliferation and dissemination. In addition, some microelements and metabolic syndrome (MetS) are closely related to the aggressive behaviours of PTC.

Hu et al. reported that alterations in neural biological processes contribute to the formation of the PTC microenvironment [183]. In PTC tissues, different types of neurons, such as cholinergic, dopaminergic, and serotonergic neurons, classified by neuronal markers, can play modulatory roles in microenvironment formation. For example, the cholinergic marker SLC18A3 is positively correlated with activated CD8^+^ T cells, effector memory CD8^+^ T cells, and T-cell trafficking, whereas the dopaminergic marker SLC6A3 and serotonergic markers show negative correlations with these T-cell changes [183] (Fig. 5).

#### Cancer-associated fibroblasts

Cancer-associated fibroblasts (CAFs) constitute the main cell type in the tumour stroma. During the recruitment and transformation of tumour cells, normal fibroblasts transition into activated CAFs, augmenting tumour progression [184]. In direct cell-cell contact and indirect mechanisms, tumour cells induce nonmalignant cells, such as fibroblasts, endothelial cells, and immune cells, to migrate to the TME [185].

A recent study revealed that stromal CAFs accumulate at the invasive front of PTC tumours, where the deposition of collagen (Col1) and the expression of lysyl oxidase (LOX) enzymes were also detected, confirming the significant association between these factors [186]. Col1 is the most abundant ECM scaffolding protein, and its deposition in the TME is related to tumour progression and metastasis. Jolly et al. proposed a regulatory loop between thyroid tumour cells, CAFs, collagen, and Lox [187]. CAFs are recruited to the thyroid TME in Braf^V600E^/Pten^−/−^/TPO-Cre thyroid cancer models, which stimulates CAFs to upregulate Col1 [185]. Moreover, tumour cells crosslink the CAFs derived from Col1 fibres in the TME by upregulating Lox, leading to a stiffer Col1 matrix, which promotes tumour cell motility and progression [185] (Fig. 5).

#### Tumour-associated neutrophils

Neutrophils account for ~70% of the circulating leucocytes in the human body, participate in the inflammatory response and represent the first line of defence against extracellular pathogens. Tumour-associated neutrophils (TANs) perform various functions in cancer. For example, they can sustain tumour angiogenesis and promote tumour cell proliferation and dissemination. Additionally, their antitumour functions have been shown to stimulate an adaptive immune response and secrete cytotoxic mediators to render tumour cells more susceptible [187, 188]. N6-methyladenosine (m6A) is the most common RNA modification in eukaryotic cells [189]. He et al. reported that METTL3 downregulation was associated with poor prognosis in PTC patients [190]. The downstream m6A targets of METTL3 were sequenced as c-Rel and RelA. YTHDF2 belongs to the largest family of m6A reader proteins that specifically recognise c-Rel and RelA. YTHDF2 can assist METTL3 in modulating c-Rel and RelA mRNA stability, leading to the activation of the NF-κB signalling pathway. Downregulation of METTL3 increases the secretion of interleukin-8 (IL-8) in PTC cells and recruits TANs to create an immunosuppressive tumour microenvironment to promote PTC progression [190] (Fig. 5).

#### Inflammation in PTC

Abundant evidence shows that PTC has an inflammatory TME [191]. Inflammation appears to be linked to tumorigenesis. Several studies suggest a strong association between chronic inflammation and neoplastic transformation associated with PTC development [192]. When inflammatory cells release highly reactive nitrogen and oxygen species, repeated tissue damage and regeneration of tissue may cause permanent genomic alterations in proliferating epithelium, including point mutations, deletions, or rearrangements, and induce neoplastic transformation [193]. In addition, activated immune cells in the TME secrete proinflammatory cytokines and chemokines that promote tumour cell proliferation [194]. The inflammatory microenvironment and its components regulate the initiation and development of PTC malignant behaviours.

In chronic lymphocytic thyroiditis, such as HT, the microenvironment is infiltrated with lymphocytes and other immune-competent cells, and some soluble mediators, including chemokines and cytokines, are closely related to cellular transformation and PTC progression [195].

In addition, rearrangements of the RET receptor (RET/PTC), RAS and BRAF, among other oncoproteins, can activate a proinflammatory state and upregulate several chemokines (e.g. CCL2, CCL20, CXCL8/IL-8, and CXCL12), the chemokine receptor CXCR4, and cytokines (e.g. IL-1B and GM-CSF) in PTC [194]. These secreted chemokines or cytokines in the TME act as oncogenic triggers to activate PTC invasion and metastasis or malignant growth. For example, the inflammatory cytokines TNFα and CXCL10 in the TME can be taken up by PTC cells, decreasing the binding between the transcription factor EGR1 and the lncRNA LNCPTCTS promoter to reduce the expression of LNCPTCTS. As a tumour suppressor, LNCPTCTS binds eEF1A2 and promotes the interaction between eEF1A2 and SNAIL, but decreased LNCPTCTS induces the expression of SNAIL in the nucleus and then inhibits the expression of E-cadherin and PEBP1 to activate EMT and MAPK signalling to enhance the progression and invasion of PTC cells [196].

Furthermore, clinical evidence shows that a higher blood neutrophil-lymphocyte ratio (NLR), which is an indicator of the systemic inflammatory response in patients with tumours, is positively correlated with larger tumour size and a greater risk of recurrence in individuals with thyroid cancer [197]. Moreover, targeting inflammation could serve as a potential approach for aggressive PTC treatment. Blocking the NF-κB pathway activated IL-8 secretion via the small molecule BAY 11-7082 in PTC cells, and the IL-8 antagonist SB225002 could eliminate chemotaxis for tumor-associated neutrophils in the TME of PTC to inhibit PTC growth and metastasis [190]. However, little is known about the regulatory effects of inflammation on the malignant behaviours of PTC. The identification of the precise mechanisms by which inflammatory factors drive the aggressiveness of PTC is of great future value.

## Conclusion

PTC is the most common endocrine malignancy, and its incidence has been rapidly increasing. As the most common pathological subtype of thyroid cancer, PTC has an excellent prognosis and survival outcome compared with rare but highly malignant ATC, PDTC, or MTC. Although PTCs grow slowly in situ, especially PTMC, they still exhibit highly aggressive characteristics, including aggressive variants, capsular infiltration, ETE, CLNM and even distant metastasis. More importantly, these aggressive characteristics increase the risk of tumour recurrence.

In the clinic, active surveillance has gradually become an alternative choice for slowly growing PTCs, but there is no doubt that PTCs possess aggressive characteristics and need timely surgical intervention in the early stages or comprehensive treatment in the late-advanced or metastatic stages. For PTCs with highly aggressive phenotypes, radical surgical resection should be performed in a timely manner. For preoperative evaluation, the location of the PTC tumour lesion within the thyroid gland; its spatial relationship with the thyroid envelope and surrounding tissues, such as the anterior cervical muscle, trachea, recurrent laryngeal nerve, and parathyroid glands; and cervical lymph node metastasis should be accurately assessed to guide a reasonable surgical scope. This may include thyroid lobectomy or total thyroidectomy with or without lymphadenectomy or adjacent organ resection, and it may be performed as open surgery, endoscopic surgery, or robotic surgery [198–200]. During surgery, attention should be given to radical resection and lymph node dissection, and to the identification and protection of adjacent tissues and organs, such as the recurrent laryngeal nerve and the parathyroid glands. If necessary, intraoperative neuromonitoring and identification of the parathyroid glands should be applied for functional protection during surgery, especially for patients undergoing laparoscopic surgery or robotic minimally invasive surgery [198, 199, 201].

However, because of insufficient understanding of factors driving the aggressiveness of PTC, some highly aggressive PTCs do not receive timely treatment and develop into late-advanced or metastatic stages. Therefore, it is necessary to understand the characteristics of the aggressiveness of PTC, reveal the underlying molecular mechanisms driving aggressiveness, and provide guidance for precise therapy for patients with more aggressive tumours.

How to recognise and diagnose the high aggressiveness of PTC in the early stages remains a challenge in clinical practice. A better understanding of the epidemiology, metastatic features, risk factors and molecular mechanisms underlying tumour behaviour is helpful for developing new clinical applications, including preoperative diagnosis and postoperative follow-up strategies, novel biomarkers and better molecular targeted therapy. PTC cell invasion does not depend on a single characteristic or mechanism. Multiple molecular mechanisms related to various risk factors are involved in this process. This review summarises the risk factors and mechanisms of PTC cell invasion and provides an overview of the novel concept of highly aggressive PTC.

The high aggressiveness of PTC develops from multiple regulatory levels, from the cellular network to the tumour microenvironment. EMT has been a hot topic in recent cancer studies, including those on PTC. There is still much work to be done to understand the connection between EMT and signalling pathways. Metabolic reprogramming of tumour cells is another issue associated with PTC invasion. The PI3K/AKT, MAPK, STAT3 and Wnt/β-catenin pathways are among the most common signalling pathways that drive PTC progression and metastasis, which indicates that novel diagnostic and therapeutic mechanisms to target these pathways in highly aggressive PTC should be developed. However, PTC cell metastasis involves more than a single pathway, and factors such as DNA methylation, histone acetylation, chromatin accessibility, noncoding RNA regulation, and the TME, including innervation, CAFs, neutrophils, and several inflammatory factors, are also associated with PTC cell metastasis. Therefore, complex and comprehensive studies are needed in the future to reveal the complex mechanisms of PTC for the preoperative diagnosis of aggressive behaviour, appropriate risk stratification, customisation of the most suitable and individualised targeted therapy, and therapy response tracking.

Overall, awareness of the high aggressiveness of PTC, identification of the molecular mechanisms driving the development of highly aggressive PTC, identification of biomarkers for the prediction and diagnosis of highly aggressive PTC, and identification of potentially efficient targets for aggressive PTC are urgently needed in an effort to provide a basis for the precise treatment of PTC.
